# Searching for Electrical Properties, Phenomena and Mechanisms in the Construction and Function of Chromosomes

**DOI:** 10.5936/csbj.201303007

**Published:** 2013-06-27

**Authors:** Ivan Kanev, Wai-Ning Mei, Akira Mizuno, Kristi DeHaai, Jennifer Sanmann, Michelle Hess, Lois Starr, Jennifer Grove, Bhavana Dave, Warren Sanger

**Affiliations:** a Human Genetics Laboratory, Munroe-Meyer Institute for Genetics and Rehabilitation, University of Nebraska Medical Center, Omaha, Nebraska, 68198-5440, USA; b Department of physics, University of Nebraska at Omaha, Nebraska, 68182, USA; c Applied Electrostatics Laboratory, Department of Environmental and Life Sciences, Toyohashi University of Technology, Tempaku-cyo, Toyohashi, Aichi, 441-8580, Japan

## Abstract

Our studies reveal previously unidentified electrical properties of chromosomes: (1) chromosomes are amazingly similar in construction and function to electrical transformers; (2) chromosomes possess in their construction and function, components similar to those of electric generators, conductors, condensers, switches, and other components of electrical circuits; (3) chromosomes demonstrate in nano-scale level electromagnetic interactions, resonance, fusion and other phenomena similar to those described by equations in classical physics. These electrical properties and phenomena provide a possible explanation for unclear and poorly understood mechanisms in clinical genetics including: (a) electrically based mechanisms responsible for breaks, translocations, fusions, and other chromosomal abnormalities associated with cancer, intellectual disability, infertility, pregnancy loss, Down syndrome, and other genetic disorders; (b) electrically based mechanisms involved in crossing over, non-disjunction and other events during meiosis and mitosis; (c) mechanisms demonstrating heterochromatin to be electrically active and genetically important.

## Introduction

Our search for electrical properties, phenomena and mechanisms in the construction and function of chromosomes are based on the satellite associations of the acrocentric chromosomes in plants, animals and humans. It is known that associations develop only among charged satellites, but the origin and nature of the interactive forces responsible for satellite charge and association formations remain unknown. For the last 30 years, we have tried to find a reasonable explanation for the origin and nature of the interactive forces responsible for charging satellites leading to satellite associations.

The first attempt failed to reveal an explanation through experimental studies with chromosomes of parasitic worms of the classes Trematoda, Nemadoda and Cestoda [1–5].

The second attempt involved extensive studies on the satellite associations among human acrocentric chromosomes, such as those found to be present in increased numbers in families with Down syndrome. These studies also failed to provide a reasonable understanding [6–7].

The third attempt also failed to find a rational explanation during our studies on satellite associations observed in human cancers, infertility, pregnancy loss, and other diseases and disorders with chromosomal origin [8–11].

Given lack of success with classical methods of chromosomal studies, an untraditional approach aimed at comparing the structure and function of chromosomes with objects in biology, physics, chemistry and other disciplines which exhibit similar construction and function has been developed. These comparative studies revealed that chromosomes are amazingly similar in their construction and function to electrical transformers [12]. These similarities are quite unusual and difficult to interpret solely from a medical and biological point of view. These findings were presented for critical examination and laboratory test confirmation by physicists from the labs in USA and Japan. Results from this joint interdisciplinary work that involved specialists in genetics, molecular biology, medicine and physics are presented in this publication.

## Materials and Methods

Searching was completed on both experimental studies and literature data. The chromosomal and DNA studies were completed in four separate laboratories from the USA, Japan and Bulgaria.

In the Human Genetics laboratory at the University of Nebraska Medical Center, Munroe-Meyer Institute for Rehabilitation and genetics in Omaha, Nebraska, USA, chromosomes from humans and animal specimens were studied since 1972. The materials and methods used in these studies include classical and modern technologies for genetics and molecular biology.

High resolution chromosome analysis was performed on lymphocytes from peripheral blood using G-banding procedures following cell culture with mitogens. Numerical and structural chromosome aberrations with diagnostic and prognostic implications in bone marrow, cancer blood, lymph node, solid tumor, product of conception (POC), amniotic fluid (AF), skin, liver and other tissue specimens. Karyotyping was completed using CytoVision Software version 4.5.2 and Leica microsystems.

Fluorescence *in situ* hybridization (FISH) studies were performed on metaphase and interphase cells utilizing probes for centromere enumeration, deletions/duplications, gene rearrangements and translocations.

Molecular microarray procedures were performed utilizing genomic DNA extracted from peripheral blood, amniotic fluid (AF), product of conceptions (POC), tumor specimen. Specimen DNA was hybridized with normal reference DNA to the oligonucleotide copy number platform. Postnatal 180,000 (180K) oligonucleotide array (Agilent Human Genome CGH 180K customized array, hg19) was used for targeting known clinically relevant genes implicated in human developmental disorders at the exon level (2-10Kb) to 50Kb whole-genome coverage.

Chromosome breakage studies were performed on peripheral blood lymphocytes from patients having an increased incidence of chromosome breakages. Additional cultures were established in the presence of Mitomycin C (MMC) and Diepoxybutane (DEB) to observe chromosomal breakage and were exposed to X-rays. The cells were analyzed using G-banded techniques for the number and type of structural chromosomal abnormalities (lesions per cell).

Protocols, reports, and images from all studies were kept in paper charts until June, 2010, after which they have been retained in the Soft Cytogenetic Computer program (SCC) along with all images of analyzed cells and karyotypes. Re-examinations and repeated studies could be performed using all studied slides and cell pellets, which are stored in the laboratory for future use. Cells pellets from blood studies, touch preparations from whole tissue, and portions of whole specimen are also frozen and stored for future studies.

Physical and statistical methods were used from Purdue University Department of Physics on experimental studies of the latticed dynamics and refined force constants to construct the dynamic matrices of long-chain DNA double helix molecules which derived their vibrational frequencies and eigenmodes. The materials and methods used in these studies are described in previous publications [13–15].

The real-time observation of electrostatic forces involved in enzyme transport along a single strand of DNA has been studied experimentally in the Applied Electrostatics Laboratory, Toyohashi University of Technology, in Japan. Materials and methods used in these studies are published by [16–17].

Experimental studies on the construction and function of chromosomes from parasitic worms of the class Trematoda, Nematoda and Cestoda have been completed at the Bulgarian Academy of Sciences, Sofa, Bulgaria at institute, known today as the Institute of Experimental Morphology and Pathology. The materials and methods used in these studies are described by [1–5].

All photomicrographs of chromosomes are taken with microscope “Opton” in magnifications x1000.

## Results and Discussion

### 1. Electrical similarities of chromosomes

Similarities between chromosomes and electrical transformers are found at three levels: in construction, effects and defects.

#### 1.1. In construction

**Figures 1, 2 and 3:**
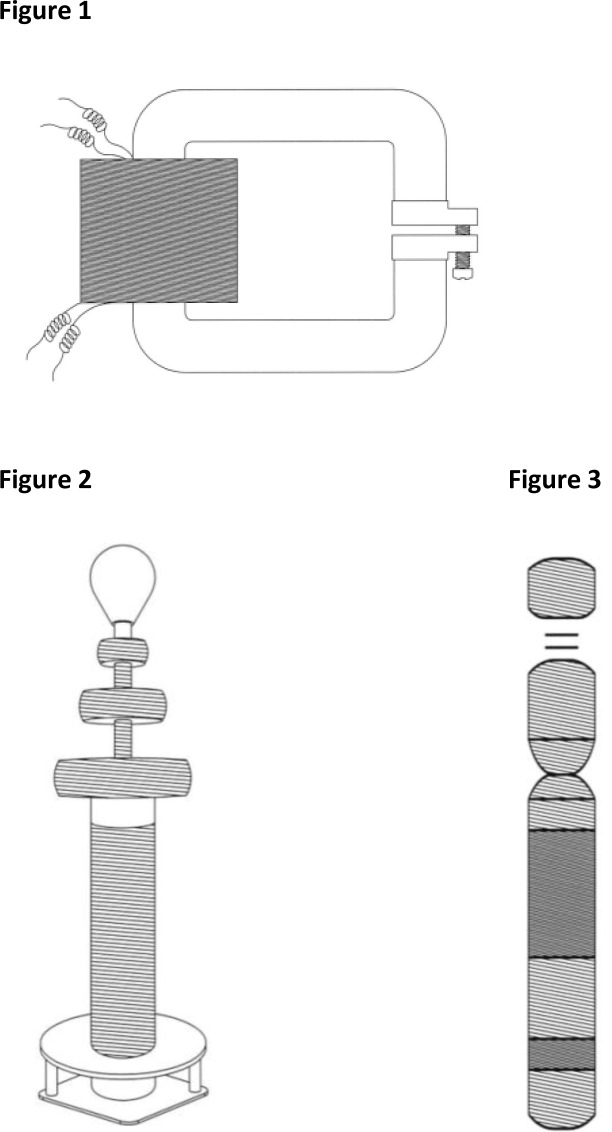
Graphic illustration of a conventional transformer of Stanley (1), a Tesla coil transformer (2), and a human chromosome 21 (3) enlarged to match the size of Figure 2. In its tightly coiled and highly repetitive rich in heterochromatin areas (illustrated in black) chromosome 21 of Figure 3 is similar in construction to the tightly coiled windings of the conventional transformer of Figure 1. In its loosely coiled areas (illustrated with lines) human chromosome 21 is similar in construction to the loosely coils of the Tesla coil transformer of Figure 2.

Chromosomes and transformers are amazingly similar in their construction, sharing a similar principal of organization. Both are constructed of long, coiled, string-shaped material. The building material of a Tesla coil and conventional transformer is metal wire with specific requirements for its conductivity and packing ratio. In a conventional transformer, the coils are very tightly coupled over the iron core (Figure 1) and voltage gain is determined by the number of turns in the coils. This works well at normal voltages but is inconvenient for high voltage operations. In a Tesla coil transformer, the windings are loosely coupled, with large air gaps between the windings (Figure 2). As a result of this construction, the primary and secondary coils typically share smaller magnetic fields.

In chromosomes, the building material is DNA, known as chromatin, which is coiled and packed with the support of histone and non-histone proteins. Chromatin is divided into two types, euchromatin and heterochromatin (Figure 3). Euchromatin is loosely coiled, similar to the windings of a Tesla coil transformer. Heterochromatin is tightly coiled, similar to those found in a conventional transformer.

Interestingly, Tesla [18] invented his transformer about 60 years before Watson and Crick [19] discovered the double helix structure of DNA and about 90 years before the first official idiograms and illustrations of the shape of human chromosomes were published in the first edition of An International System for Human Cytogenetic Nomenclature (ISCN). Yet, as shown in Figures 2 and 3, his transformer is amazingly similar in shape and in principal construction to that of human chromosome 21.

The reasoning for the construction of both conventional and Tesla coil transformers is well understood. However, the purpose of the shape and construction of chromosomes remains speculation. For many years it was believed that the only purpose of tightly coiled DNA was to fit chromosomes into the small cell nucleus. Recently it was discovered that the coiling and uncoiling of DNA plays important roles in gene expression and regulation. A new branch of genetics called epigenetics was created to study the heritable changes in gene expression or cellular phenotype caused by mechanisms other than changes in the underlying DNA sequence. Based on the results presented below in the text, we hypothesize that perhaps a chromosome's coils play another important role connected with energy or power.

#### 1.2. In effects

In both conventional and Tesla coil transformers, different effects are known to occur after being connected to an electrical current. For example, the Tesla coil is well known for its unique ability to act as a magnifying transmitter, in which it discharges high frequency energy that is visible and safe under specific conditions. Additionally, the Tesla coil possesses the phenomenal ability to transmit signals and power from one transformer to another simply by magnetic flux, without using wires. This phenomenon was demonstrated by Tesla in many different ways, including lighting an electrical bulb in his hand without direct connection to the Tesla coil transformer sitting several meters away.

Chromosomes can be charged. This is known from the time when Ruzicka [20] used silver staining to study structures (nucleolar organizing areas, NORS) inside of the cell nucleus. Heitz [21] discovered that in packed chromosomes NORS are located on the p-arm of the acrocentric chromosomes in regions known as satellites and stalks. Creighton and McClintock [22] identified a phenomenon known today as satellite association. These associations occur when charged satellites of two or more chromosomes are attracted. However, the origin and the nature of the energy responsible for these charges, attractions and associations are unknown and not well understood.

#### 1.3. In defects

In the conventional transformer, one type of defect can occur when the winding of the coils are too closely packed and tightened over the iron core. In such conformation the primary and secondary windings share 80-90% of their magnetic fields, which caused the insulation between the two sets of windings to break down, causing electrical sparks, cracks, splits, bursts, breaks, and fusions.

In chromosomes, similar gaps, breaks, fusions, and other defects occurred in areas rich in tightly coiled, highly repetitive heterochromatin. In human chromosomes, such areas are located in the centromeres and telomeres of all chromosomes, in the satellites of acrocentric human chromosomes 13, 14, 15, 21, and 22, and in the variable *qh* regions of chromosomes 1, 9, 16 and Y. The most common and severe damage to chromosomes occurs at the same areas rich in tightly coiled, highly repetitive heterochromatin.

The similarities between chromosomes and electrical transformers, as described above, have gone unrecognized for more than 120 years, because historically there has been communication gap between the scientific disciplines of physics and biology. In part this gap may be due to the different methodologies and purposes in the respective fields, but it may also be due to unshared professional knowledge, skills, and experience across disciplines.

### 2. Electrical components of chromosomes

Chromosomes display structures and functions equivalent to the main electrical components.

#### 2.1. Electric generator

An electric generator is a device that converts mechanical energy to electrical energy, which pushes electric charge (usually carried by electrons) to flow through an external electrical circuit system. There are many kinds of mechanical systems that facilitate electric generation, such as reciprocating or turbine steam engines, water falling, internal combustion engines, windmills, hard cranks, and compressed air.

In complicated molecules like chromosomes, low-lying excited states, such as vibrational motions take place at each chemical bond, like those between hydrogen and other atoms in the base pairs of DNA, with frequencies ranging from microwave to infrared. In turn, these motions cause the charge on molecules to move, producing local electrical currents. According to Gigliardi [23], generating energy from DNA and chromosomes is very complicated. He suggested that the level of energy generated depends on factors, such as the chemical components of the DNA, physical configuration of its coils, level of pH and of amount of Ca+ and other ions inside the nucleus. Local electrical current may be generated in different places and at different base pairs, but is not isolated because the base pairs are arranged in circuits. Through these circuits, electrical currents develop in two or more different places and can communicate in polarizing form to act as a positive and negative charge.

#### 2.2. Electric charge

Electric charge is an intrinsic physical property of matter that causes an experience of force when near other electrically charged matter. Two types of electric charges exists: positive and negative. Basic law of physics state that similar charges repel each other and opposite charges attract each other. Electric charge is also fundamentally conserved at the subatomic level, which determines the electromagnetic interaction. Accelerating charges produce electromagnetic fields that govern by basic law of physics, such as the Maxwell's equations [24] and the Newton's law of motion [25].

In chromosomes, the same positive and negative types of electric charges exist on a nano-scale. For nearly 60 years, the concept of “charge” has been widely used in cytogenetic in the description of satellite associations of acrocentric chromosomes. In the last decade it has been used to describe events at the DNA and chromosome level in terms of charge equilibration [26]; charge transduction [27]; charge transport [28]; charge migration [29]; and chromosome motions [30].

#### 2.3. Conductor

A conductor is a material that contains mobile electric charges and allows them to move rather freely. In all metals, the movable charged particles are electrons. Positive charges may also be mobile, but they are much heavier and slower than when compared to electrons. Conducting materials include metals, electrolytes, superconductors, ionic conductors, semiconductors, plasmas and some non-metallic conductors, such as graphite and conductive polymers.

In DNA and chromosomes, 21^st^ century experiments with atomic force microscopy (AFM) and other modern technologies demonstrate the various phenomena related to the motion of electric charges: electron attachment [31]; electron transfer [32]; electron migration [33]; electron tunneling [34]; and ion motions [35]. In these experiments, DNA was found to have the characteristics of an electric conductor. In some experiments DNA was found to act as a semiconductor [36] or as a superconductor [37].

#### 2.4. Capacitor (Condenser)

A capacitor (sometimes known as condenser) is a passive two-terminal electrical component used to store energy in the form of an electric field. When there is a potential difference (voltage) across the conductors, a static electric field develops across the dielectric, causing positive charge to collect on one plate and negative charge on the other plate; hence the energy is stored in the electrostatic field.

Bodily tissue, including chromosomes are known to act as a capacitor (condenser) since the time of Tesla [38] who suggested that:*“Bodily tissues are condensers or capacitors, perhaps with innate compatibility toward the presence of high electric fields which already is present in cellular tissue, which is the basic component (dielectric) for an equivalent circuit only recently developed for the human body.”*

#### 2.5. Switches

A switch is an electrical component that can break an electrical circuit, interrupting the current by on or off positioning or diverting it from one conductor to another.

In chromosomes, the same on-off switch functions have been reported several times. The first one is connected with the discovery of Barr and Bertram [39] and Lyon [40] Barr body, lyonization, dosage compensation and the inactivation of the X-chromosome. The second time the on-off switching process is reported at the chromosome level by Riggs *et al* [41] in epigenetic, where the heritable changes in gene expression or cellular phenotype is caused by mechanisms other than changes in the underlying DNA sequence. The third time the on-off switches in DNA and at the chromosome level was reported in the Encyclopedia of DNA Elements (ENCODE) for the human genome, where 22,000 genes are working under the control of 4,000,000 switches [42]. It is also known that, various enzymes with electrical charges are transported on DNA for replication and other functions. At the replication point, where DNA strands separate to form single strands, charged enzymes could be stopped or derailed from the DNA strand. This could be regarded as a “switch”. The derailed charged particles could form a space charge, which would attract DNA molecules near the space charge. This attractive force could cause DNAs to attach.

#### 2.6. Transmitter and receiver of light and optical and or acoustic vibration

Transmitters and receivers are electrical devices that transmit or receive light and other types of electromagnetic waves, which are then converted into usable form, such as information transfer. This occurs by converting the waves into tiny alternating currents, which are applied to a receiver that extracts the desired information, using filters to separate the wanted frequency signals from all other signals, and then amplifies the power of the signal. The information produced may be in the form of light, images or digital signals.

In studies on DNA and chromosomes, the emitted light is known as ”biophotons”, “mitogenetic radiation” and “Gurvich ray” as reported by Gurvich [43–44]. Sound and image generation were related to holograms and biocomputers, as reported by Peter Gariaev *et al* [45]. Information and transmission properties of viral RNA were discussed by Nobel Prize winner Luc Montagnier at al. [46].

#### 2.7. Transformer

A transformer is a power converter that transfers electrical energy from one circuit to another through inductively coupled conductors, the transformer's coils. A varying current in the *primary* winding creates a varying magnetic flux and thus a varying magnetic field through the *secondary* winding. This varying magnetic field induces a varying electromotive force (EMF), or “voltage”, in the secondary winding. When the windings are tightly coiled, transformers develop cracks, splits, bursts, breaks, fusions, and other problems which are caused by the increased level of electromagnetic forces.

In DNA and chromosomes, the same type of structural defects like cracks, splits, bursts, breaks, fusions, and other problems occur in places where the chromatin, known as heterochromatin, is tightly coiled and highly repetitive. Furthermore, the regions of tightly coiled and highly repetitive heterochromatin are the places where attractive, repelling, resonance, fusion and other forces are known to exist in DNA and chromosome.

#### 2.8. Electric current

Electric currents are composed of moving charges that produce an electromagnetic field in the nearby vicinity. The strength of the electromagnetic field directly depends on the magnitude of the current.

In DNA and chromosomes electric currents can be associated for the exchange of electrons and ions as described above. As previously described (section 2.5), electrically charged enzymes are transported along the DNA macromolecule, therefore their motion can be considered a production of “electric currents”. We suggest that the electrical impedance mismatch might be one of the possible mechanisms for increasing the current in DNA and at the chromosome level. Such impudent mismatching may exist between the homologous chromosomes of mother and father. When these homologous pairs meet in the embryonic cells, mother's and father's chromosomes may be composed of different amounts of heterochromatin. In such cases, the chromosome possessing the larger amount of heterochromatin produces stronger electrical current and acts as an electron donor. The homolog of smaller heterochromatic variant and weak electrical current acts as a recipient accepting the transferred electrical current. If the electrical current between the donor and recipient chromosome is very strong and becomes violent it may cause breaks, inversions, translocations, fusions and other abnormalities. Some of these abnormalities that can be associated with cancer, infertility, mental retardation, spontaneous abortion and other clinically important diseases of chromosomal origin.

#### 2.9. Electrical grid

An electrical grid is an interconnected network for delivering electricity from suppliers to consumers. In the power industry, electrical grid is a term used for an electricity network that has three distinct operations: 1) electrical generation 2) electric power transmission 3) electric power distribution.

In DNA and chromosomes a similar interconnected network with the same three components exists. It is demonstrated by the satellite and telomere attractions and associations involving multiple chromosomes organized in an effective electrical network. In certain circumstances this network can indicate defects as breaks, translocations, and fusions involving two or more chromosomes. In all cases, multiple chromosomes can be seen to communicate and act together, controlled by electromagnetic forces, which may be responsible for the development of breaks, translocations, attractions, associations and fusions of chromosomes.

### 3. Electrical forces and phenomena at the chromosome level

The similarities, of components and properties as seen in chromosomes and electrical systems are not isolated or co-incidental events. Several types of electrical phenomena are found to exist in chromosomes, which can be divided into several groups, based on their locations and expression.

#### 3.1. Attractive and repulsive forces and phenomena

Based on their location, some forces and phenomena are described as satellite associations, joint formations, telomeric associations, and ring formations.

##### 3.1.1. Satellite associations and joint formations

**Figures 4-6:**
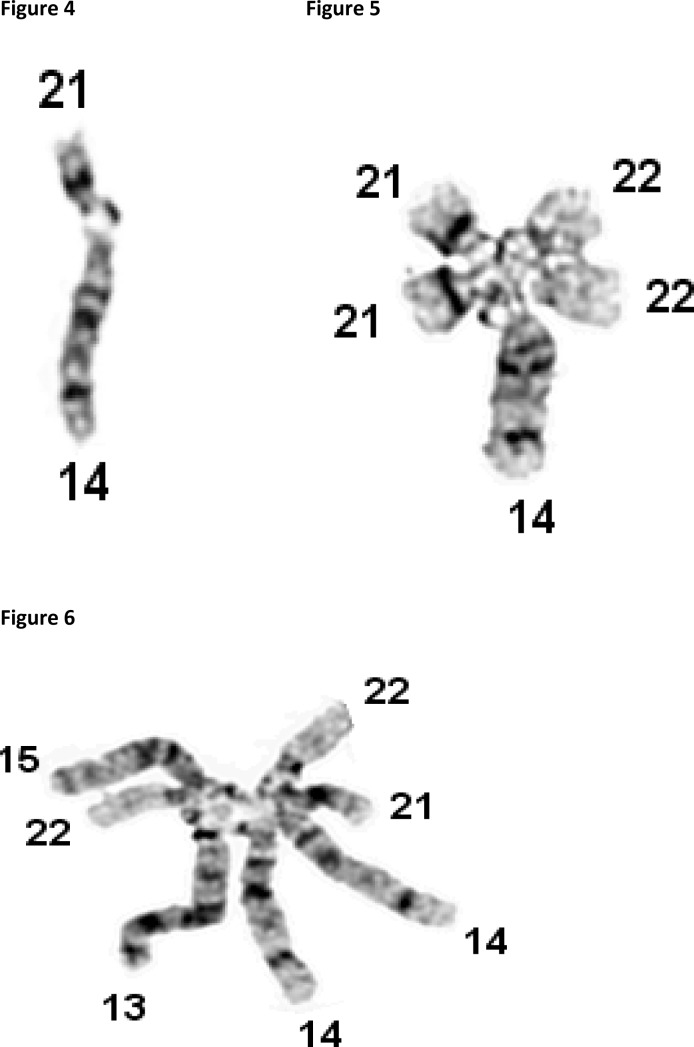
Microphotographs of satellite associations involving: Two chromosomes 14 and 21 (Figure 4). Five chromosomes 14, 21, 21, 22 and 22 (Figure 5). Seven chromosomes 13, 14, 14, 15, 21, 22 and 22 (Figure 6).

Satellites, located on the short arm of acrocentric chromosomes of humans, animals and plants were described by Heitz [21]. Typically satellites are oval in shape, dark staining structures, rich in tightly coiled, highly repetitive heterochromatin. Satellite associations in human acrocentric chromosomes were first described by Ferguson-Smith and Handmaker [47] and Ohno *et al* [48]. In humans, satellite associations are formed between charged satellites of the acrocentric chromosomes 13, 14, 15, 21 and 22. The most common associations are formed between two acrocentric chromosomes, as shown in Figures 4. Occasionally, enlarged associations involving three and more chromosomes can be found in configurations like those shown in Figures 5 and 6. In our studies, extensive satellite associations have been found in patients with Down syndrome, infertility and spontaneous abortions.

**Figures 7-9:**
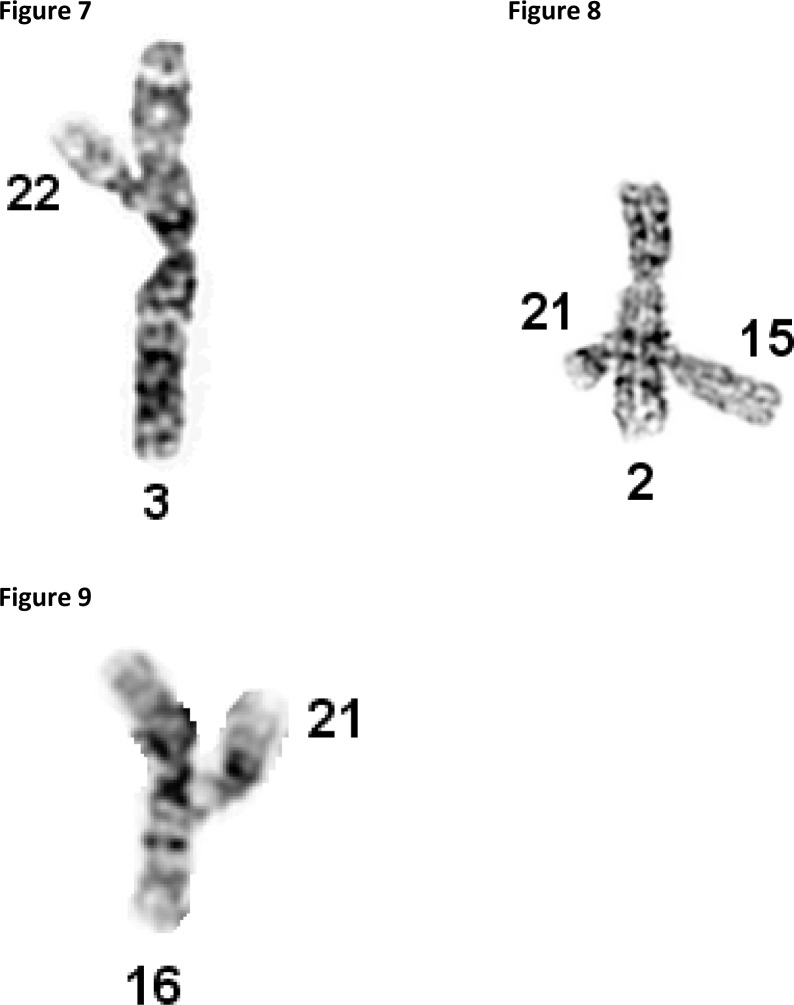
Microphotograph of satellite joint formation between: Satellites of small acrocentric chromosome 22 attached (associated) to the short arm of chromosome 3 (Figure 7); Satellites of acrocentric chromosomes 15 and 21 attached (associated) to the long arm of chromosome 2 (Figure 8); Satellites of chromosome 21 attached (associated) with the centromere region of chromosome 16 (Figure 9).

Joint formations are found between the satellites of acrocentric chromosomes and the dark bands of chromosomes, which are rich of tightly coiled, highly repetitive heterochromatin. (Figures 7–9).

The mechanism responsible for the formation of satellite associations and joint formations has been unexplainable for many years, in part because, these phenomena are not considered significant, not studied in detail, and not compared with similar phenomena seen in physics and electrical engineering. Our collaborative studies across disciplines reveal that the mechanisms responsible for the formation of satellite associations and joint formations are similar to these described in physics by Maxwell's equations. Even the terminology used in physics and genetics is similar. For example “charge” is widely used in physics for the description of attractive and repulsive forces of electromagnetic origin. “Charge” with unspecified definition is used in clinical genetics for the description of satellite associations like these shown in Figures 4–6 [49].

**Figures 10 and 11:**
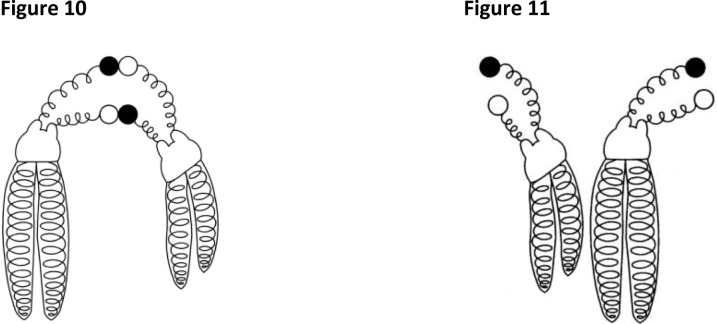
Free hand drawing of two chromosomes which are: Pointing their satellites toward each other indicating an attractive force of opposite (negative and positive) charges, as predicted by the Maxwell equations (Figure 10). Satellites orientated in opposing directions indicating a repulsive force generated from two like (positive or negative) charges (Figure 11).

Charged satellites demonstrate at the nano-scale level, attractive and repulsive forces which are similar to these described by Maxwell equations (Figures 10 and 11).

In clinical genetics, attractions and satellite associations, like these of Figure 10 are found in nearly all of the chromosome complements from lymphocytes from peripheral blood samples. During the last 30-40 years many authors have reported an increased incidence of such satellite associations in the peripheral blood of patients with Down syndrome [50], Turner syndrome [51], and infertility [52]. Attempt were made to establish some type of correlation between the satellite associations and development of these disorders, however all of these efforts failed because satellite size, shape and associations did not provide an explanation for observations of the electrical mechanism and forces responsible in the development of satellite associations and joint formation.

The correlation between satellite associations and disorders do exist but have gone unrecognized since the mechanism is based on energy, which is invisible using present day genetic methods and technologies. However, these forces are studied in detail and well known in physics and electrical engineering. In Maxwell's equations, it is indicated that the attractive forces of electromagnetic origin develop from the transfer of electrons, ions and other particles. These transfers create current, which develop electromagnetic fields and forces. These electromagnetic forces could cause cracks, breaks, fusions and other defects in electrical systems when the winding of the coils is too tightly packed, just is seen to occur in phenomena of a conventional transformer. According to these phenomena, the electromagnetic forces of a conventional transformer developed specific types of defects when the winding of the coils is too closely packed and tightened. This happens because the primary and secondary windings of such constructions share 80-90% of their magnetic fields and the insulation between the two sets of windings have broken down, causing electrical sparks, cracks, splits, bursts, breaks, and fusions.

We suggest that a similar electrically based mechanism is responsible for the development of satellite associations among acrocentric chromosomes. In support of this suggestion is the fact that satellites, like the conventional transformer, are built from tightly coiled heterochromatin.

###### 3.1.2. Telomeric association and ring formation

Telomeric associations and ring formations are found in chromosomes of humans, animals, and plants. They involve telomeres, which are rich in tightly coiled, highly repetitive heterochromatin. The first telomeric associations and ring chromosomes were discovered by Lilian Morgan [53–54]. The mainstream concept developed by Counter *et al* [55] suggested that telomeric associations and ring formations develop as a result of two telomere ends joining after becoming damaged or dysfunctional. It is generally believed that the mechanism that joins the ends occurs as a result of the detachment of protective proteins from the chromosome ends. This concept was fitting for those cases where telomeres had two breaks, one from each arm of the chromosomes involved, where the breaking of two telomere ends is capable of fusing damaged arms together. However, this concept does not fit well for telomeric associations and ring formation in which one or two ends of chromosomes are intact and free of damage.

Furthermore, this concept is based on proteins only and does not provide an appropriate explanation for many of the constitutional and acquired telomeric associations and ring formation events. For example, in constitutional telomeric associations, usually only telomeres of two chromosomes are involved, while acquired associations are more complex, often involving more chromosomes. In most cases, the constitutional ring formations are simple and monocentric, while acquired rings are more complex, larger in size, more variable in construction, and much more unstable in nature as they form polycentric rings. It is still unknown why ring formations are rare in benign tumors, common in certain invasive tumors and very common in certain subgroups of sarcoma and carcinoma. Finally, the mechanisms responsible for the complex rearrangements leading to segmental microdeletions and microduplications in ring chromosomes are not understood.

**Figures 12-14:**
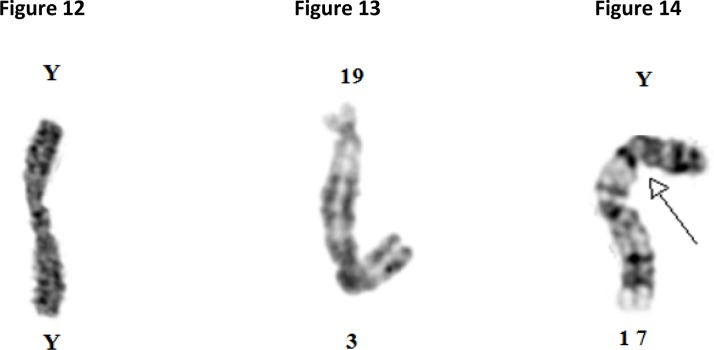
Microphotograph of human chromosomes with telomeric associations involving the long arm of chromosome Y (Figure 12); long arms of chromosome 3 and 17 (Figure 13); and short arm of chromosome Y and 17 (Figure 14).

This protein-based concept fails to explain how, why and what kind of forces are responsible for the attraction and orientation of different chromosomes and the fusing of telomeres permanently, like these shown in Figures 12–14.

**Figures 15-19:**
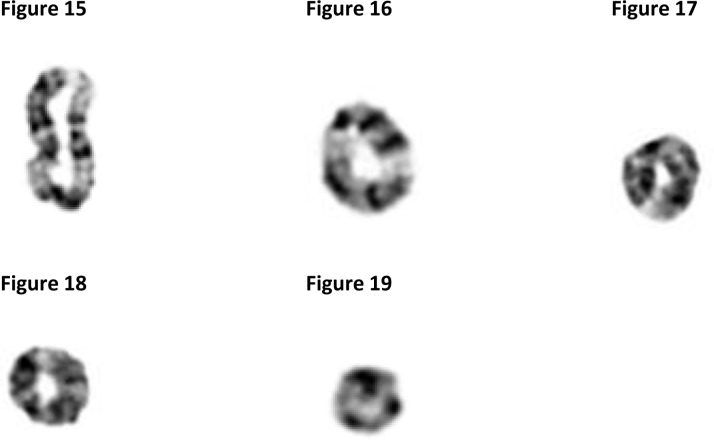
Microphotographs of ring chromosomes 9 (Figures 15 and 16) and X chromosomes (Figsures 17–19).

The protein-based mechanism cannot provide an appropriate explanation why, how, and the kind of forces that are responsible for twisting, banding and fusing chromosomes permanently in their telomeres like these shown in Figures 15–19.

We suggest that the mechanisms responsible for the development of telomeric associations and ring formations are similar to those described for the satellite associations and joint formations. In support of this suggestion is the fact that telomeres, like satellites, are rich in tightly coiled, highly repetitive heterochromatin, which would explain the strong attractive electromagnetic forces that exist in these chromosome areas.

#### 3.2. Mechanism based on resonance, vibrational and oscillatory forces

Resonance, vibrational and oscillatory forces and their mechanisms are well known in physics, yet they are unrecognized at the chromosome level in genetics.

In physics, electrical, and mechanical engineering, it is well known that resonance forces occur with all types of vibrations or waves: mechanical, acoustic, electromagnetic, nuclear magnetic, and electron spin resonance. It is also known that vibration is a mechanical phenomenon whereby oscillations occur about the equilibrium positions of the system where the total energy is the lowest. The motion is repetitious in both space and time, has a definite frequency and number of cycles and is intrinsic on the mechanical properties of the system and amplitude, which is often small compared with the size of the system. Resonance oscillation takes place when the system is interacting with a time dependent external disturbance with a similar frequency that renders a self-reinforced vibration and causes the amplitude to grow and reach the size of the system. Eventually, in conditions with strong resonance, oscillation and vibration forces could get out of control and become violent, leading to “self-destruction”. For example, in nonlinear mechanics it is known that the normal vibration, resonance and oscillated forces could become violent and disasterous, causing cracks, breaks and other damages. Accordingly, resonance disaster occurs as a result of repeated storage and additional energy input in the system until its load limit is exceeded causing these self-destructive forces to occur. In some cases, these forces could be very strong and capable of destroying buildings and bridges made from steel and stone. This happened to Tacoma Narrows Bridge in Tacoma, Washington, USA, when it collapsed on November 7, 1940, through resonance generated from strong winds. The Broughton Suspension Bridge collapsed in England on April 12, 1831, by resonance induced by soldiers marching over the bridge in step. Marching solders also brought disaster to the Angers Bridge in France when it collapsed on April 16, 1850, while a battalion of soldiers marched across it, killing over 200 of the troop.

If the resonance, vibrational, and oscillation forces are capable of destroying solid constructions like steel and stone bridges, then they would be capable of causing cracks, breaks and other destructive forces to the fragile construction of DNA and chromosomes. Based on data from quantum mechanical studies on the molecular structures of DNA and chromosomes, we can confidently regard DNA and chromosomes as mechanical structures on a microscopic scale, which are anticipated to obey the same laws of physics as other mechanical structures. It seems very promising to evaluate the corresponding resonance, vibrational, and oscillatory forces, operational in DNA and chromosome structures, as well as their ability to cause cracks and breaks.

**Figures 20-23:**
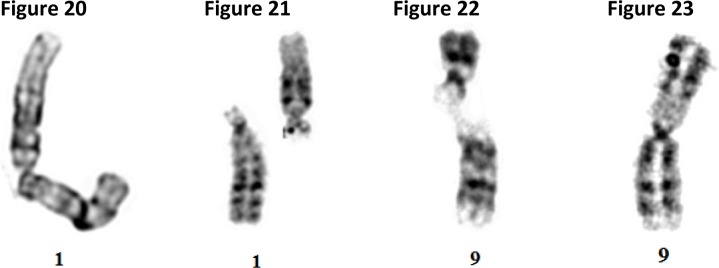
Microphotograph of: chromosome 1 with a gap in the centromere region (Figure 20); chromosome 1 broken into two pieces at the *qh* area (Figure 21); chromosome 9 with broken q arm at the *qh* region (Figure 22): chromosome 9 with two breaks followed by repulsion, attraction and fusions forces forming a pericentric inversion (Figure 23).

We suggest that the multi-level layers of tightly coiled heterochromatic areas, particularly those consisting of centromeres and secondary constriction *qh* areas of human chromosomes 1, 9, 16, and Y, experience similar self-destructive forces in the form of cracks, breaks, inversions, translocations and fusions like those in Figures 20–23.

**Figures 24-28:**
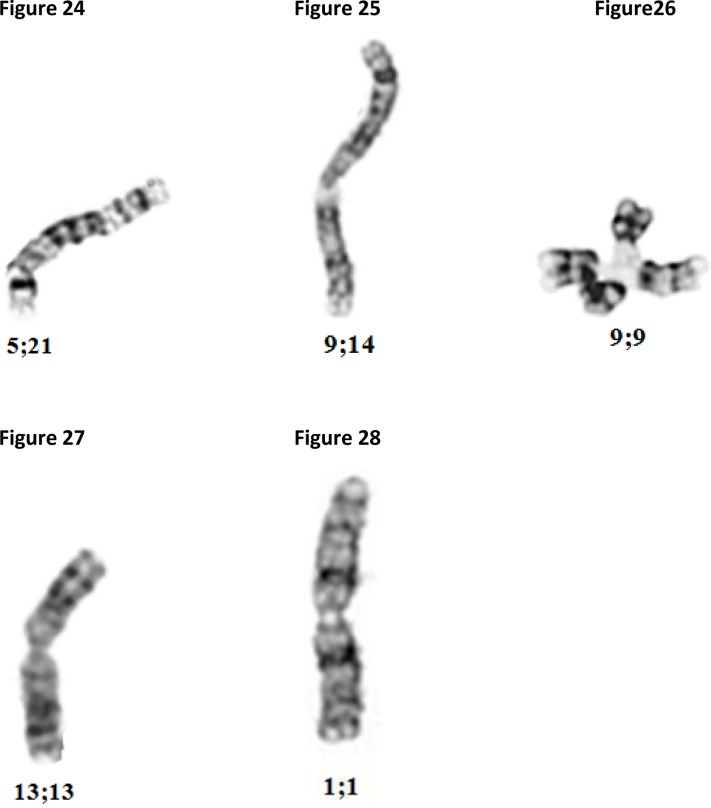
Microphotograph of human chromosomes: translocation involving the long arms of chromosomes 5 and 21 (Figure 24) and chromosomes 9 and 14 (Figure 25). Fusion of two chromosomes 9 at the *qh* region (Figure 26), two long arms of chromosomes 13, (Figure 27) and two long arms of chromosome X (Figure 28).

More severe breaks, translocations and fusions involving two chromosomes are shown in Figures 24–28.

**Figures 29-32:**
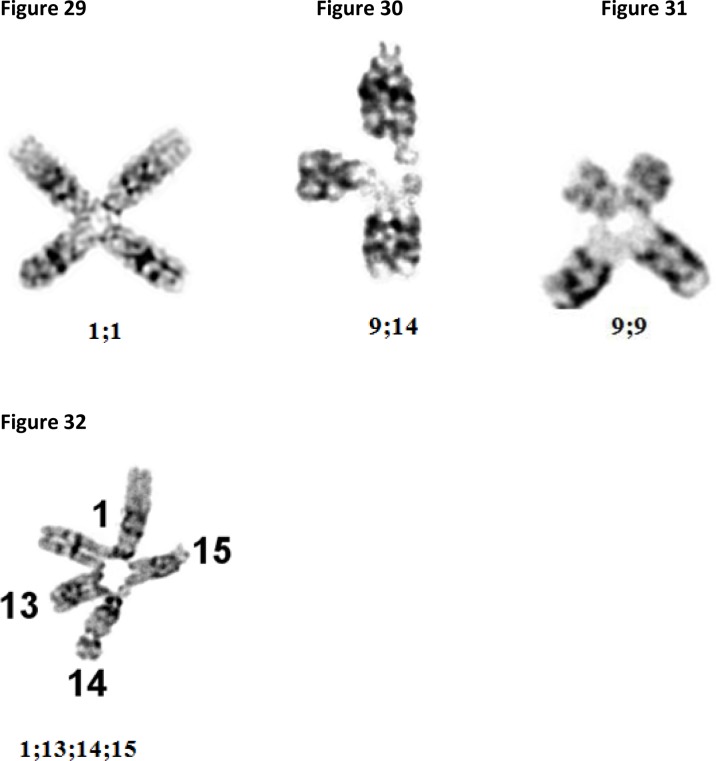
Microphotographs of: Two chromosomes number 1 exposed to DEB showing fusion in *qh* areas (Figure 29). Chromosome 9 broken in *qh* area and fused with long arm of chromosome 14 after exposure to DEB (Figure 30). Two chromosomes 9 fused in *qh* areas after exposure to MMC (Figure 31). Chromosomes 1, 13, 14 and 15 melted and fused after exposure to X-ray (Figure 32).

Extreme damage, such as the fusions shown in Figures 29–32 are found in chromosomes exposed to X-ray and toxic chemicals, typically known as DNA crosslinkers, such as Depoxibutane-1,2;3,4 (DEB), and Mitomicin-C (MMC).

Cracks, breaks, inversions, translocations and fusions of chromosomes like these shown in Figures 20–32 were discovered in experimental genetics about a century ago, when in 1910 Tomas Morgan and his co-workers started extensive studies of chromosomes from fruit flies of genus *Drosophila*. Their research led to the discovery that X-rays cause cracks, breaks, inversions, translocations and fusions of chromosomes. However, the mechanism responsible for the development of these phenomena was unknown. Nearly 60 years later, scientists noticed that chromosomes, breaks, inversions, translocations and fusions are associated with the development of cancer in humans, particularly for the chromosomes 1, 9, 16, and Y, with larger *qh* areas [56–58]. Some scientists [58] suggest that correlation exists between the development of cancer and heterochromatic variants of chromosomes, however the mechanism responsible for such correlations is not known.

We suggest that such correlations and mechanisms exist, but have gone unrecognized since they are based on resonance, vibration, oscillation, attraction, fusion and other forces at the chromosome level, which are undetectable with current methodologies in clinical genetics. Examined using modern physics methods and technologies, these forces and mechanisms have become known to exist in DNA and at the chromosome level.

#### 3.3. Other forces and mechanisms found in DNA and RNA at the chromosome level

During the last 20-30 years, with the development of new methods and technology, transport-related forces and mechanisms have been extensively studied in DNA and chromosome material.

##### 3.3.1. Forces that move enzymes along DNA

The electrical current along DNA molecules can be expressed in terms of transport characteristics of electrons, ions and charged bio-molecules, such as enzymes and nucleotides, subject to specific forces.

Enzymes work systematically during DNA replication at the replication site and have to be transported, possibly along the DNA strand.

**Figure 33:**
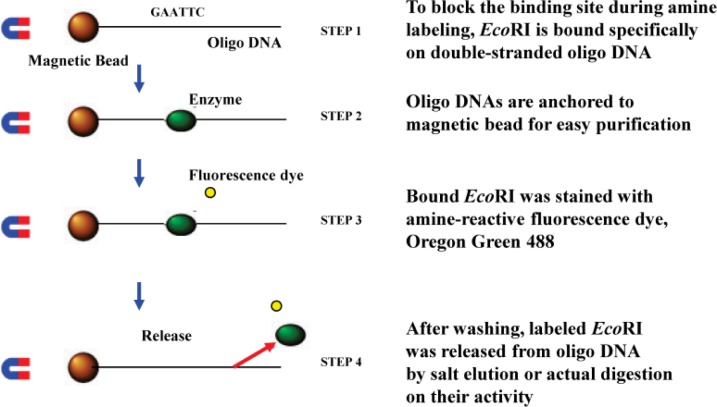
Concept of *Eco*RI staining without blocking the activity

**Figure 34:**
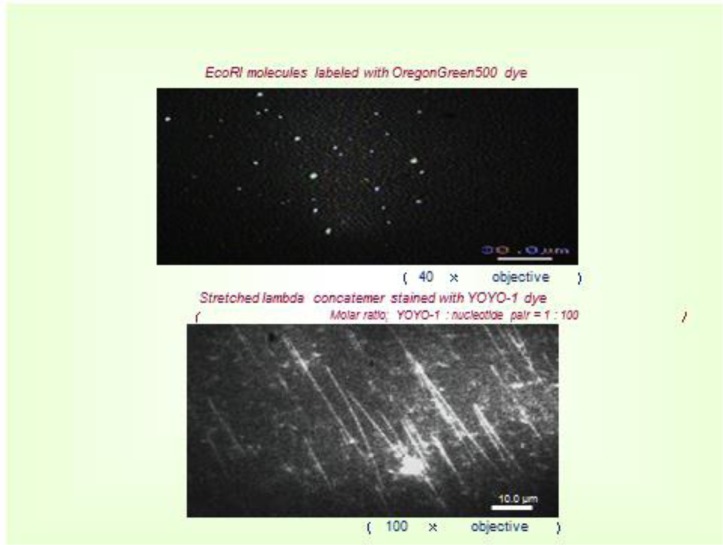
Fluorescent Image of *Eco*RI molecules and stretched DNA.

An interaction between a restriction enzyme and a DNA strand was observed using fluorescent microscopy [16]. The restriction enzyme, *Eco*RI, was fluorescently labeled with OregonGreen500 with the staining method depicted in Figures 33. 3-5 lambda phase DNA strands were connected to form a concatemer with a length of about 50-100 micro-meters. These concatemers were the fixed onto a glass plate treated with 3-Aminopropyltriethoxysilane on the surface, so that the those having a negative charge were loosely attached with a stretched form, as shown in Figure 34. The concatemers were fluorescently labeled by YOYO-1 for observation. For observation of the movement of *Eco*RI, the concatemers were not labeled, and magnesium ions in the solution were completely removed to avoid cutting DNA molecules. The solution used was: 50 mM Tris-HCl (pH 7.5), 100 mM NaCl, 7 mM 2-Mercaptoethanol.

**Figure 35:**
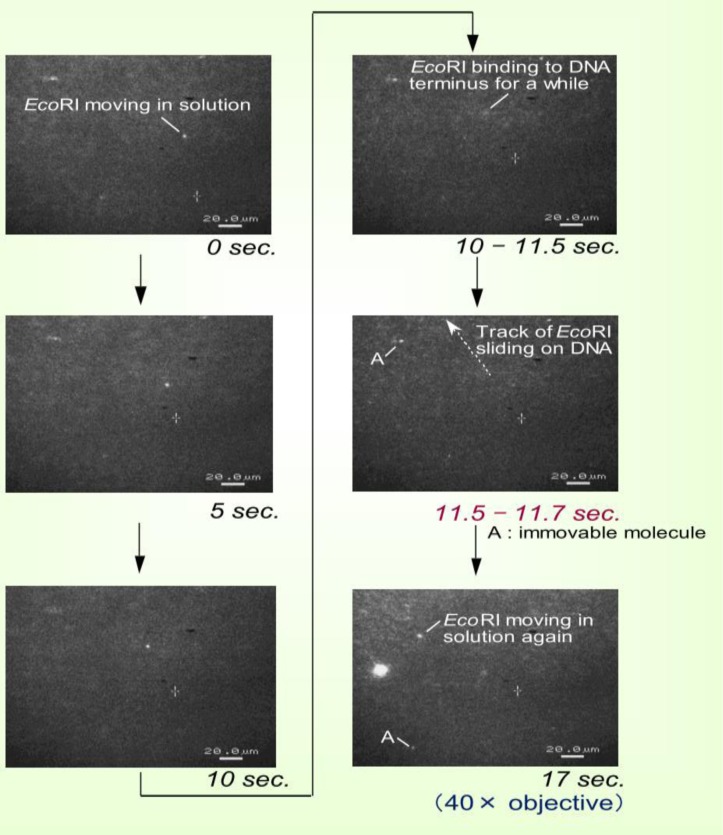
Direct observation of sliding of *Eco*RI on DNA under a fluorescence microscopic field

The solution of *Eco*RI was added to the glass plate with the stretched concatemers where *Eco*RI migratation was seen along with the laminar flow of the solution. The movement of the *Eco*RI was recorded, as shown in Figures 35. At 10-11.5 seconds drifting movement of the *Eco*RI stopped for about one second. During this period, the image of EcoR1 became slightly out of focus, but wasadjusted. *Eco*R1 might be attracted to a concatemer to recombine. Then at 11.5-11.7 seconds, *Eco*R1 moved linearly towards the upward left of the glass for 50 micro-meters within 0.1 second. The velocity again decreased and drifted with the flow. The quick linear motion was the sliding of the *Eco*RI on the DNA strand. Velocity of the sliding motion was about 500 micrometer/second, or 1.5×10^6^ bp s^-1^. These results were similar to those obtained by other methods in test tubes [59]. There are three different types of diffusion, such as one-dimensional “sliding” with non-specific binding, three-dimensional diffusion with association and dissociation with DNA, and intersegment transfer [59]. In this experiment, one-dimensional sliding was occurring [60–62]. The *Eco*RI did not recognize the restriction site, possibly due to the overstretch by the force of flow from B-type (pitch: 3.38nm to S-type pitch: 4.34nm). It should be noted that the direction of the sliding along the stretched DNA is determined by chance (Figure 33).

The experimental observation indicated that an enzyme can be bound to DNA and can be moved with approximately 500 micrometer/second velocity. The direction of the initial sliding is random; however, one sliding particle should push the other charged particle with the same polarity. This interaction could eventually give one directional movement of charged particles with the same polarity. In this case, polarity of the charge of the *Eco*RI is positive since it was attracted by the negatively charged DNA. Charge quantity cannot be estimated. We assume that an enzyme, or any binding protein, has an electric charge of ten units and that those charged particles exist 50 nm apart (or larger than Debye length), and that they are sliding on a DNA molecule with a velocity of 500 micrometer/second in one direction. Current flowing along DNA is calculated to be 1.6 x 10^-15^ A. This current flows along a DNA strand attached around a histone of 11 nm diameter, the magnetic flux density could be, B = 0.4 x 10^-12^ T, assuming that the number of turns in unit length is 2x10^8^ turn/m. This magnetic flux could cause an electromagnetic force of an order of 10^-30^ N on a DNA carrying 1.6 x 10^-15^ A. This electromagnetic force can affect the adjacent histone-DNA complex to resume its regular form (Figure 34).

At the point of replication, where the strand becomes single, the current along a DNA strand could not flow as smoothly as the double stranded part since the binding site of enzymes is specific to double stranded DNA. In this case, the charge should accumulate at the point of replication or the charge should detach and be released into media. If 1/10 of the charged particles moving along a DNA molecule are stacked and/or released at a locus, 1.6 x 10^-16^ C is accumulating in one sec.

There is a diffusion of the charge to disappear and the net charge existing at that point is determined by the balance of the charge sliding into the point and the diffusion. If we assume the charge quantity to be 1.6 x 10^-16^ C, this could cause significant force to the other DNA strand close to the point. Electric fields at a distance (r) from the point can be calculated as E(r) = 1.4 x 10^-6^/r^2^ V/m. If r = 100 nm, then E = 1.4 x 10^7^ V/m. In this electric field, DNA strands are affected significantly. For instance, in 1,000 V/m electric fields, double stranded lambda DNA can be stretched in liquid [17]. The electric field around the point of replication may assist enzymes, nucleotides, and other materials necessary to drive DNA to the point of replication. Crossing of genes could also take place randomly by chance if this large electrostatic force attracts other parts of the DNA strand.

###### 3.3.2. Charge transfer mechanisms that recognize mismatch and repair damaged segments of DNA

The previous extensive studies [63–68] have shown that damaged or mismatched bases in DNA could be repaired by the base excision repair enzymes (BER) that replenish the defective base. Many of the molecular structures of BER were investigated thoroughly, but the mechanisms that explain how BER finds lesions are still not completely understood. One possible explanation is the charge where the electrons are freed by recently absorbed BER enzyme travel along the DNA strands. The charges bounce back and forth multiple times between the DNA and enzyme, while being absorbed or re-emitted by lesions and guanine radicals in the vicinity. Stochastic models were developed and computer simulations were performed on these processes. Insights of the mechanism, if revealed, will offer further understanding regarding the electrodynamics of the charge transfer mechanisms seen in DNA.

####### 3.3.3. Forces that move chromosomes during cell division

Using several well-tested physical and biochemical methods, studies have now established that electrostatic forces take part in the arrangement and movement of chromosomes in the spindle apparatus during cell division [23, 30, 69–82]. Gagliardi [78] argued that these forces induce electrostatic charges in mitotic chromosomes residing at kinetochores, centrosomes and on chromosome arms. He suggests that these electrostatic forces develop as a result of cellular charge distribution, which contains several charge contributions: the positive charge of kinetochores, the negative charge of centrosomes, the positive charge of molecules that bind microtubules with the kinetochore, the pH level, and the amount of Ca+ and other ions in the nucleus during mitosis. Based on his studies and if the arguments supporting our model are correct, it is believed that after the eventual discovery of the specific molecules in kinetochores and centrosome matrices and the mitotic chromosome motion will be disclosed. Of course, detail microscopic molecular dynamics simulations are necessary in order to prove this theory. Nevertheless, what is indisputable is that the electrostatics forces between the charges are responsible for the post-attachment chromosomes movement.

The latest development and innovative work of Zhao and Zhan provides a systematic approach to the understanding of the physical mechanism for chromatin oscillation cluster (COC) formation and long-distance chromatin interactions. In their papers [83, 84], they regard the super-macromolecular complexes as the most essential factor in the transcription of eukaryotic cells. They recognize that oscillation synchronization and clustering of different chromatin regions allow efficient, systemic, genome-wide regulation of transcription. Furthermore, they propose that that electric fields generated by synchronized oscillation of microtubules, centrosomes, and chromatin fibers expedites major activities during mitosis and meiosis, including centrosome trafficking, chromosome assembly in mitosis and synapsis between homologous chromosomes in meiosis. We find their methodology perceptive and meaningful, when combined with ours, which stimulate further insight in genomic research.

######## 3.3.4. Charge distribution forces of large molecules

Molecules are composed of many different atoms with equal amount of positive and negative charges. Although the entire molecule is neutral, the molecule is defined by a complex charge density determined by quantum mechanics and laws of electrodynamics (i.e. Maxwell's equations). The molecules are interacting via these charge distributions, which in principle can be explained in terms of a few well-established equations; however, solving the equations is an insurmountable task. Systematic ways of approximated approach have been developed by generations of physicists which are constructed and applied to different types of molecules, from simple diatomic molecules to complicated long-chain DNA and protein molecules. For example, GAMESS (The General Atomic and Molecular Electronic Structure System), [http://www.msg.ameslab.gov/gamess/capabilities.html] is one of the current most commonly used quantum chemistry packages that unite quantum mechanics and lattice dynamics to compute charge densities of the gigantic molecules, which drive intermolecular interactions, especially protein-ligand and DNA base pair interactions. Molecular dynamic simulation is performed experimentally to explain these observations [85–86]. This methodology is a frequently adopted scheme in biomedical material research.

######### 3.3.5. Warburg hypothesis: energy and forces generated from metabolism

Metabolically generated energy and forces in living cells described by Warburg and Negelein [87], Warburg [88], Krebs and Henseleit [89], Krebs and Johnson [90] and many other scientists, are different in origin from the electrical properties, phenomena and forces of chromosomal origin described in this paper. In the Warburg hypothesis and Warburg effect, Warburg [88] suggests that the replacement of the respiration of oxygen in normal body cells by a fermentation of sugar is the prime cause of cancer. According to his hypothesis cancer, malignant growth, and tumor growth are caused by the fact that tumor cells mainly generate energy (as e.g. adenosine triphosphate-ATP) by glycolysis, a non-oxidative breakdown of glucose. This is in contrast to “healthy” cells which mainly generate energy from oxidative breakdown of pyruvate, which is an end-product of glycolysis, which is oxidized within the mitochondria. Based on this concept, Warburg suggests that the cancer cells are driven from a decrease in mitochondrial respiration.

Grander [91], Bertram [92] and other scientists suggest that the metabolic change observed by Warburg is not so much the cause of cancer, as he claimes, but rather, it is one of the characteristic effects of cancer-causing mutations. According to these scientists cancer is caused by mutations and altered gene expression, in a process called malignant transformation, resulting in an uncontrolled growth of cells. In clinical genetics, malignant transformations of cancer cells are demonstrated by chromosome aberrations, such as breaks, deletions, translocations, fusions and other defects [93]. However, the mechanisms responsible for the development of these and other chromosomal aberrations are not understood.

We believe that the electrical properties, phenomena and mechanisms described in this publication could provide a better understanding of the forces of energy responsible for development of chromosomal aberrations in cancer cells considered to be the primary cause of cancer.

########## 3.3.6. Speculations on long-range interactive forces

The speculations for long-range interactive forces at the DNA level belong to Dr. Peter Gariajev, renowned Russian theoretical physicist and his co-workers [94–98], who proposed the notions of “DNA quantum biocomputers”, “DNA phantom effect” and “Wave-based genetics”, suggesting DNA could: (1) carry out distant (multi-kilometer) transfers of genetic/metabolic information in the form of special physical fields; (2) introduce this information into human biosystems; and (3) perform strategic management functions concerned with biosystems, biochemical systems, and actual physiological conditions.

Similarly, Nobel Prize laureate Luc Montagnier (Medicine, 2008) claimed that DNA could send “electromagnetic imprints” of itself into distant cells and fluids, which can then be used by enzymes to create copies of the original DNA [46]. This information can be used for transmission properties of viral RNA from one cell to another through teleportation [46].

We find these imaginative and speculative ideas channeling new thoughts into biological and medical sciences. Further studies are necessary for their prove and confirmation.

## Conclusions

The presented data lead to several conclusions listed below:

Chromosomes and transformers are amazingly similar in their principles of construction and functional effects and defects.

The structural components of chromosomes corresponded with the components of electrical transformers (i.e. generators, chargers, conductors, capacitors (condensers), switches, transmitters, receivers, transformers, currents, and electric grids).

The functions of chromosomes display electromagnetic interactions and phenomena, such as attractions, repulsions, vibration, resonance, breaks, and fusions which are known in physics and electrical engineering; are studied using the same methods; measured with the same units for energy; obey the same physical laws and equations; and are described with the same terminology.

Heterochromatin, which comprises about 97% of the building materials of human chromosomes, has been considered to be unimportant, inert, genetically inactive, barren, non-functional and useless “junk” DNA. We suggest that heterochromatin be considered a very important area in chromosomes, actively generating and operating their own electrical forces and mechanisms.

Because of their electrical properties, and related forces and mechanisms, chromosomes should be regarded not only as vehicles for carrying genes and inheritance, but also as generators, transformers, conductors, condensers, switchers, transmitters and receivers in electric circuits, capable of operating electric currents or moving charges.

The electrical currents and mechanisms may play important roles in the essential function of chromosomes, such as gene regulation and expression; attraction, pairing and separation of chromosomes during cell division; transporting electrons, ions and other particles between DNA structures; and in moving enzymes along DNA molecules.

Occasionally, under some conditions, the electrical current of chromosomes could become violent and destructive causing cracks, breaks, inversions, translocations, fusions and other chromosomal abnormalities associated with clinically important diseases and syndromes.

Clinically important diseases and syndromes, including cancers, developmental delay, infertility, pregnancy loss, Down syndrome may be caused by violent electrical currents generated and operated by the chromosomes.

The newly discovered electrical properties, forces and mechanisms of chromosomes provide a reasonable explanation for some syndromes, diseases and conditions of chromosomal etiology, which will be described in separate publications.

The complex construction and function of chromosomes are not fully understood today, requiring time and new methodology to reveal their unique properties, function, and underlying mechanisms.
